# Low-pressure versus standard-pressure pneumoperitoneum in minimally invasive colorectal surgery: a systematic review, meta-analysis, and meta-regression analysis

**DOI:** 10.1093/gastro/goae052

**Published:** 2024-07-19

**Authors:** Justin Dourado, Peter Rogers, Nir Horesh, Sameh Hany Emile, Pauline Aeschbacher, Steven D Wexner

**Affiliations:** Ellen Leifer Shulman and Steven Shulman Digestive Disease Center, Cleveland Clinic Florida, Weston, FL, USA; Ellen Leifer Shulman and Steven Shulman Digestive Disease Center, Cleveland Clinic Florida, Weston, FL, USA; Ellen Leifer Shulman and Steven Shulman Digestive Disease Center, Cleveland Clinic Florida, Weston, FL, USA; Department of Surgery and Transplantation, Sheba Medical Center, Ramat-Gan, Israel; Ellen Leifer Shulman and Steven Shulman Digestive Disease Center, Cleveland Clinic Florida, Weston, FL, USA; Colorectal Surgery Unit, General Surgery Department, Mansoura University Hospitals, Mansoura, Egypt; Ellen Leifer Shulman and Steven Shulman Digestive Disease Center, Cleveland Clinic Florida, Weston, FL, USA; Department of Visceral Surgery and Medicine, Inselspital, Bern University Hospital, University of Bern, Bern, Switzerland; Ellen Leifer Shulman and Steven Shulman Digestive Disease Center, Cleveland Clinic Florida, Weston, FL, USA

**Keywords:** low pressure, standard pressure, pneumoperitoneum, minimally invasive surgery, systematic review, meta-analysis

## Abstract

**Background:**

We aimed to assess the efficacy and safety of low-pressure pneumoperitoneum (LPP) in minimally invasive colorectal surgery.

**Methods:**

A PRISMA-compliant systematic review/meta-analysis was conducted, searching PubMed, Scopus, Google Scholar, and clinicaltrials.gov for randomized-controlled trials assessing outcomes of LPP vs standard-pressure pneumoperitoneum (SPP) in colorectal surgery. Efficacy outcomes [pain score in post-anesthesia care unit (PACU), pain score postoperative day 1 (POD1), operative time, and hospital stay] and safety outcomes (blood loss and postoperative complications) were analyzed. Risk of bias2 tool assessed bias risk. The certainty of evidence was graded using GRADE.

**Results:**

Four studies included 537 patients (male 59.8%). LPP was undertaken in 280 (52.1%) patients and associated with lower pain scores in PACU [weighted mean difference: −1.06, 95% confidence interval (CI): −1.65 to −0.47, *P *=* *0.004, *I*^2^* *=* *0%] and POD1 (weighted mean difference: −0.49, 95% CI: −0.91 to −0.07, *P *=* *0.024, *I*^2^* *=* *0%). Meta-regression showed that age [standard error (SE): 0.036, *P *<* *0.001], male sex (SE: 0.006, *P *<* *0.001), and operative time (SE: 0.002, *P *=* *0.027) were significantly associated with increased complications with LPP. In addition, 5.9%–14.5% of surgeons using LLP requested pressure increases to equal the SPP group. The grade of evidence was high for pain score in PACU and on POD1 postoperative complications and major complications, and blood loss, moderate for operative time, low for intraoperative complications, and very low for length of stay.

**Conclusions:**

LPP was associated with lower pain scores in PACU and on POD1 with similar operative times, length of stay, and safety profile compared with SPP in colorectal surgery. Although LPP was not associated with increased complications, older patients, males, patients undergoing laparoscopic surgery, and those with longer operative times may be at risk of increased complications.

## Introduction

The use of the minimally invasive approach in colorectal surgery has been associated with decreased morbidity [1] and postoperative pain [2, 3]. Additionally, there have been many studies reinforcing the benefits of minimally invasive approaches to colorectal surgery, more recently as part of enhanced recovery after surgery (ERAS) protocols in terms of improved recovery and reductions in complications when compared with open surgery [4, 5]. One essential step of laparoscopic surgery is the creation of pneumoperitoneum, which involves inflating the abdominal cavity with carbon dioxide (CO_2_) gas to create a working space for the surgical instruments and visualization of the surgical field. The utility and safety of the use of low-pressure pneumoperitoneum (LPP) have been debated.

Some investigators have recommended using the lowest acceptable levels of pneumoperitoneum needed to maintain a safe workspace [6]. Advocates postulate that low pressure is believed to reduce the potential complications related to reduced venous return, kidney function, cardiac output, and risk of air embolus. The recent guidelines from the ERAS^®^ Society were published in 2018; since then, there have been multiple studies investigating the outcomes of LPP on surgical outcomes. Improvements in postoperative pain without increasing the length of stay (LOS), conversion rates, and complications have been demonstrated in both gynecological surgery [7] and cholecystectomy [4]. Additionally, LPP has been associated with decreased analgesic consumption in cholecystectomy with a high-to-moderate quality of evidence [8]. Díaz-Cambronero *et al.* [9] showed that the concept of a tailored approach to pneumoperitoneum with an emphasis on maintaining a lower pressure has been previously shown to be safe and feasible in colorectal surgery.

Nevertheless, while LPP has been demonstrated to be safe in multiple surgical disciplines, there is no high-quality collective evidence that LPP is safe and efficacious within the realm of colorectal surgery. In clinical practice, many surgeons continue to routinely use standard-pressure pneumoperitoneum (SPP). The hypothesis of our study is that LPP may provide short-term benefits relative to lower postoperative pain and quicker recovery while maintaining a similar safety profile to SPP. The aim of this systematic review and meta-analysis was to assess the efficacy of LPP in minimally invasive colorectal surgery for improving patient outcomes while conferring equivalent levels of operative safety.

## Methods

### Registration

The protocol of this systematic review was prospectively registered in the international prospective register of systematic reviews “PROSPERO” under the registration number CRD42023449315. There was no deviation from the registered protocol when reporting this systematic review. Reporting of the current review followed the screening guidelines established by the Preferred Reporting Items for Systematic Reviews and Meta-Analyses (PRISMA 2020) [10].

### Search strategy

Two authors (N.H. and J.D.) performed a systematic search of the literature for randomized clinical trials (RCTs) that assessed the effect of LPP on outcomes in colorectal surgery. The search process was independently conducted, after which the retrieved articles were cross-checked. If there were disagreements in regard to article selection, they were resolved by mutual agreement between the two authors. The agreed-upon list of articles was again reviewed by a third author (S.H.E.) prior to finalization.

Electronic databases, including PubMed, Scopus, Google Scholar, and clinicaltrials.gov were searched from inception through July 2023 without language restrictions. All studies other than RCTs were excluded. The databases were searched using Medical Subject Headings or the equivalent, title/author key words, truncation, and Boolean operators. Strategies included the terms “colorectal,” “surgery,” “operative procedures,” “surgical procedures,” “general surgery,” “pneumoperitoneum,” and “pneumoperitoneums.”

### Assessment of bias

Two authors (J.D. and P.R.) independently assessed the risk of bias in the studies. The revised tool to assess the risk of bias in randomized trials (ROB 2) was used to assess all RCTs [11]. Any conflicts of the interpretation of bias were reviewed and resolved by a third author (N.H.). The certainty of the evidence was graded with the GRADE approach as very low, low, moderate, or high [12]. The publication bias was considered to be assessed if 10 or more studies met the inclusion criteria.

### Data extraction and study outcomes

Two investigators (J.D. and P.R.) extracted the following information from each study:

1. Author, title, journal of publication, nct, publication year, design, setting, and country of study.
2. Number of patients in each group, age, body mass index (BMI), and sex.
3. Indication for surgery, site of surgery, and method of surgery (open, laparoscopic, robotic).
4. Primary outcomes related to efficacy including pain score in post-anesthesia care unit (PACU), pain score on postoperative day 1 (POD1), LOS, and operative time.
5. Secondary outcomes related to safety including intraoperative complications, blood loss, postoperative complications, and major complications. Major complications were classified as Clavien-Dindo grade III or greater [13].

### Data synthesis

A meta-analysis and meta-regression were conducted using EZR (Easy R) version 1.61 and the open-source, cross-platform software for advanced meta-analysis “openMeta [Analyst]™” version 12.11.14. A meta-analysis was used to calculate the weighted mean difference (WMD) in continuous variables including blood loss, LOS, operative time, and pain score in PACU. A pairwise meta-analysis was conducted to assess the difference in categorical variables including intraoperative, postoperative, and major complications using odds ratio (OR) and 95% confidence interval (CI). Statistical heterogeneity was assessed using the inconsistency (*I*^2^) statistics (low if *I*^2^ < 25%, moderate if *I*^2^* *=* *25%–75%, and high if *I*^2^ > 75%). A fixed-effect meta-analysis was used if *I*^2^ < 25% and a random-effect analysis was used if *I*^2^ ≥ 25%. Sensitivity analyses, leave-one-out analysis, and meta-regression analyses were performed. *P* values < 0.05 were considered significant.

## Results

### Description of studies and patients

**Figure 1.**
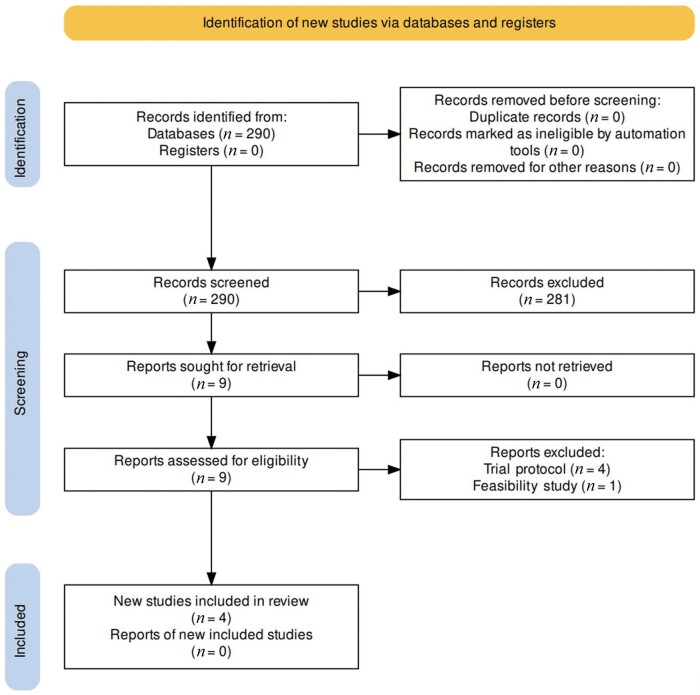
PRISMA search flowchart of this study.

After screening 290 studies, nine were initially included. Moreover, five studies were subsequently excluded as four were trial protocols and one was a feasibility study. Finally, a total of four RCTs [14–17] published between 2015 and 2022 were included in the analysis (Figure 1), three of which were from Europe and one from Asia. Two trials were multi-centric and two were single-center studies. The studies included 537 patients (male 59.7%) with a median age of 68 (range 64–68.7) years. Laparoscopy was used to perform 74.1% of the procedures, whereas 25.9% were robotic-assisted. The indication for surgery was restricted to malignancy only in two studies, whereas two studies included patients with either malignant or benign conditions. Two studies included only laparoscopic surgery and two included robotic surgery (Table 1). There were no significant differences in the sex distribution or surgery site or approach (robotic or laparoscopic) between the groups in the four included trials. LPP was defined as 5–7 mmHg [16], 8 mmHg [14, 17], or 10–12 mmHg [15]. Deep neuromuscular blockade (NMB) was utilized for LPP and moderate for SPP in two studies [14, 17] and no variation in NMB was utilized in two studies [15, 16]. Three studies included all minimally invasive colectomies [14, 15, 17], whereas one only included right or left hemicolectomy [16]. The exclusion criteria in all studies were emergency surgeries, pregnancy, patients <18 years of age, and procedures with a planned stoma formation. A search of the clinical trial registry revealed one ongoing trial, PAROS3, in recruitment (NCT 05934981).

**Table 1.** Study characteristics

Study	Country	Design	Setting	Total number of patients	Age, years, mean ± SD	Male, *n* (%)	Indication for surgery, *n* (%)		Surgical approach, *n* (%)	
							Malignancy	Benign	Robotic	Laparoscopic
Albers *et al.* (2022) [14]	Netherlands	RCT	Multi-center	178	69 ± 9	114 (64.0)	158 (88.8)	20 (11.2)	96 (53.9)	82 (46.1)
Celarier *et al.* (2021) [16]	France	RCT	Single center	127	66 ± 24	63 (49.6)	75 (59.1)	52 (40.9)	43 (33.9)	84 (66.1)
Díaz-Cambronero *et al.* (2020) [17]	Spain	RCT	Multi-center	166	68 ± 27	103 (62.0)	149 (89.8)	NA	0 (0)	166 (100)
Cai *et al.* (2015) [15]	China	RCT	Single center	66	64 ± NA	41 (62.1)	66 (100)	0 (0)	0 (0)	66 (100)

RCT = randomized controlled trial; SD = standard deviation; NA = not available.

LPP was undertaken in 280 (52.1%) patients, whereas 257 (47.8%) patients had SPP. One study [15] did not include BMI or American Society of Anesthesiologists (ASA) status. The LPP and SPP groups were comparable in terms of median age (67.3 vs 66.9 years), median BMI (26.4 vs 25.4 kg/m^2^), and ASA II–IV classification (77.7% vs 77.4%). However, the LPP group entailed a higher male sex proportion (62.9% vs 56.8%) (Table 2).

**Table 2.** Demographics and patient characteristics in the LPP group (*n *=* *280) and the SPP group (*n *=* *257).

Study	Distribution in treatment arm, *n*		Male, *n* (%)		Age, mean ± SD		BMI, mean ± SD		ASA II–IV, *n* (%)	
	LPP	SPP	LPP	SPP	LPP	SPP	LPP	SPP	LPP	SPP
Albers *et al.* [14]	89	89	57 (64.0)	57 (64.0)	68.5 ± 9.5	68.9 ± 9.2	26.2 ± 4.0	27.3 ± 4.8	67 (75.3)	70 (78.6)
Celarier *et al.* [16]	62	65	34 (54.8)	29 (44.6)	65 (20–87)^a^	67 (22–93)^a^	24.3 (16–38)^a^	23 (16–43)^a^	45 (72.6)	43 (66.2)
Díaz-Cambronero *et al.* [17]	85	81	58 (68.2)	45 (55.6)	68 (58–74)^a^	67 (59–77)^a^	27 (24–30)^a^	26.6 (23.8–29)^a^	72 (84.7)	69 (85.2)
Cai *et al.* [15]	44	22	27 (61.4)	14 (63.6)	62.6 ± 10.3	64.7 ± 6.4	NA	NA	NA	NA

BMI = body mass index; ASA = American Society of Anesthesiologists; NA = not available; LPP = low-pressure pneumoperitoneum; SPP = standard-pressure pneumoperitoneum; SD = standard deviation.

a Presented values are median (range).

### Efficacy outcomes

**Figure 2.**
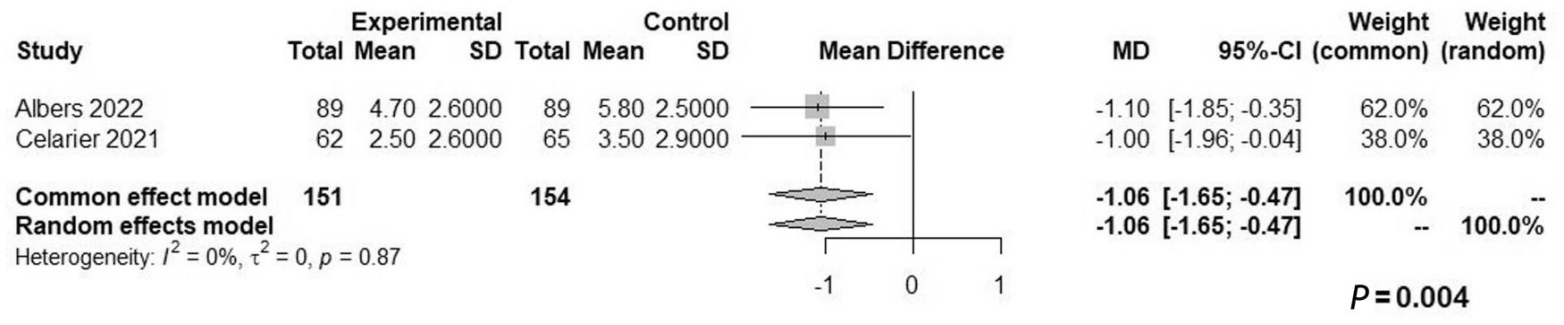
Forest plots of pain score in post-anesthesia care unit (PACU).

**Figure 3.**
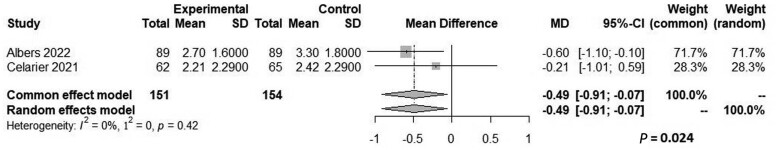
Forest plots of pain score on postoperative day 1 (POD1).

**Figure 4.**
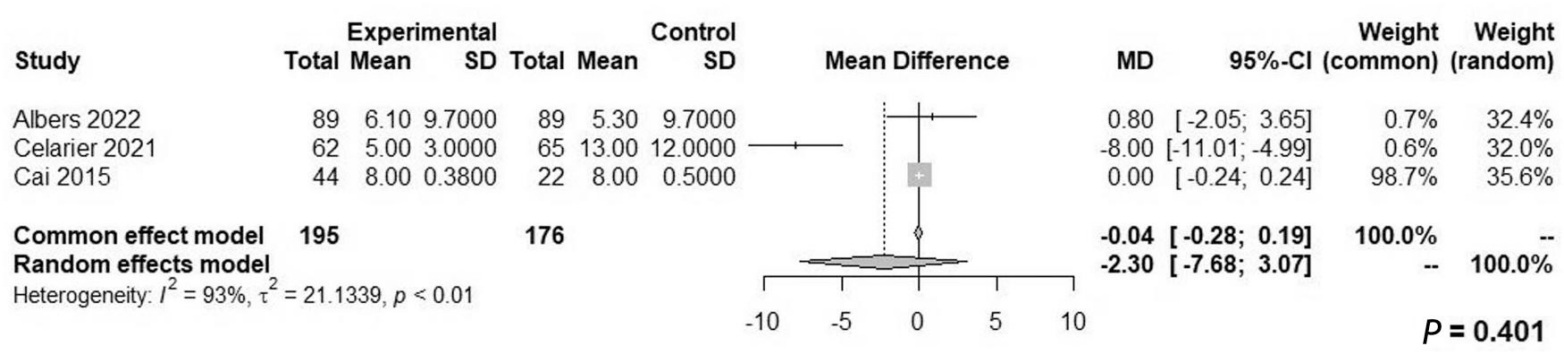
Forest plots of length of stay.

**Figure 5.**
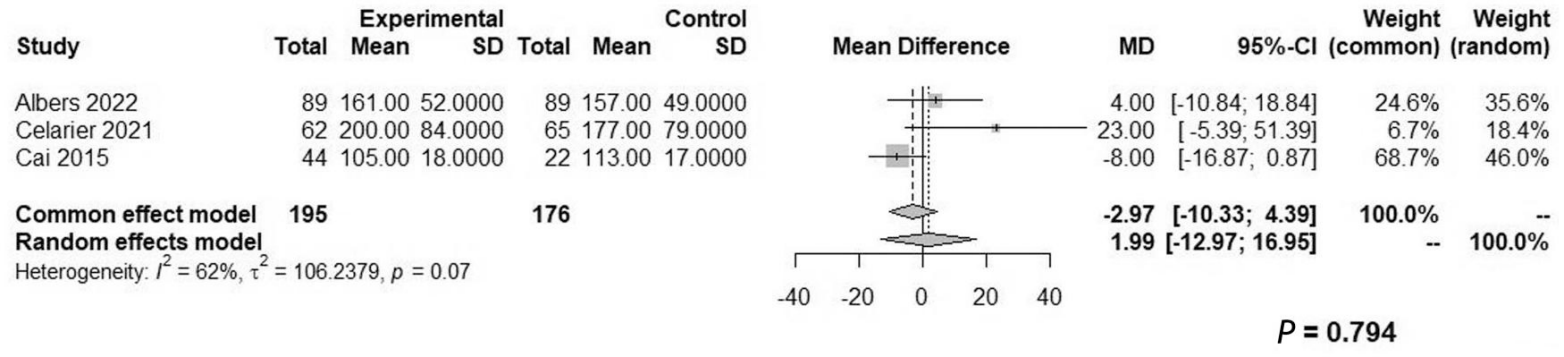
Forest plots of operative time.

LPP was associated with a significantly lower pain score in PACU (WMD: −1.06, 95% CI, −1.65 to −0.47, *P *=* *0.004, *Z* = −3.53, *I*^2^* *=* *0%; Figure 2) and on POD1 (WMD: −0.49, 95% CI: −0.91 to −0.07, *P *=* *0.024, *Z* = −2.27, *I*^2^* *=* *0%; Figure 3). There were no significant differences between the two groups relative to the LOS (WMD: −2.30, 95% CI: −7.68–3.07, *Z* = −0.84, *P *=* *0.401, *I*^2^* *=* *93%) or operative time (WMD: 1.99, 95% CI: −12.97–16.95, *Z* = 0.26, *P *=* *0.794, *I*^2^* *=* *62%) (Figures 4 and 5 and Table 3). The high heterogeneity in LOS was attributed to a significant outlier of 43 days in the control arm of one study [16].

**Table 3.** Efficacy outcomes in the LPP group (*n *=* *280) and the SPP group (*n *=* *257)

Study	Operative time (min)		Length of stay (days)		Pain in PACU	
	LPP	SPP	LPP	SPP	LPP	SPP
Albers *et al.* [14]	161 ± 52	157 ± 49	6.1 ± 9.7	5.3 ± 9.7	4.7 ± 2.6	5.8 ± 2.5
Celarier *et al.* [16]	200 ± 84	177 ± 79	5 ± 3	13 ± 12	2.5 ± 2.6	3.5 ± 2.9
Díaz-Cambronero *et al.* [17]	NA	NA	NA	NA	NA	NA
Cai *et al.* [15]	105 ± 18	113 ± 17	8 ± 0.38	8 ± 0.5	NA	NA

Presented values are mean ± SD.

PACU = post-anesthesia care unit; NA = not available; LPP = low-pressure pneumoperitoneum; SPP = standard-pressure pneumoperitoneum.

### Safety outcomes

**Figure 6.**
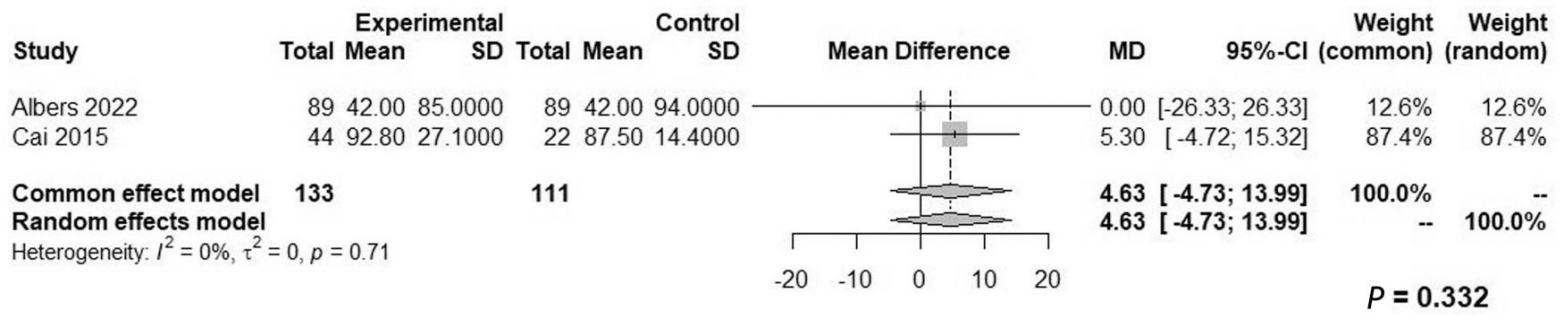
Forest plots of blood loss.

**Figure 7.**
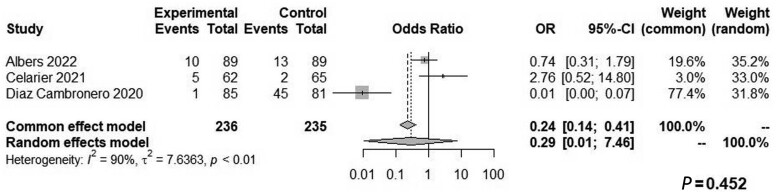
Forest plots of intraoperative complications.

**Figure 8.**
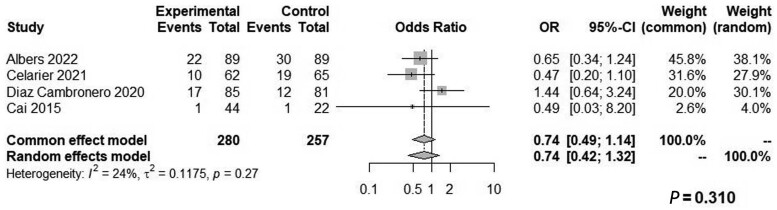
Forest plots of postoperative complications.

**Figure 9.**
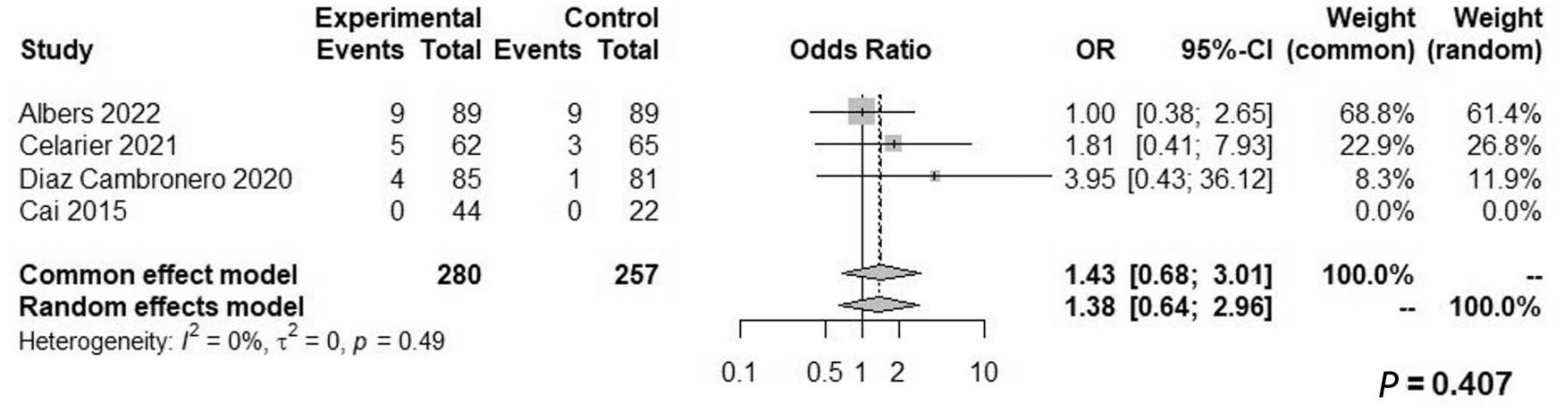
Forest plots of major postoperative complications.

Both groups had similar blood loss (WMD: 4.63, 95% CI: −4.73 to 13.99, *Z* = 0.97, *P *=* *0.332, *I*^2^ = 0%; Figure 6) and similar odds of intraoperative complications (OR: 0.29, 95% CI: 0.01–7.46, *Z* = 0.75, *P *=* *0.452, *I*^2^ = 90%; Figure 7), postoperative complications (OR: 0.74, 95% CI: 0.49–1.14, *Z* = 1.37, *P *=* *0.310, *I*^2^ = 24%; Figure 8), and major postoperative complications (OR: 1.43, 95% CI: 0.68–3.01, *Z* = −0.94, *P *=* *0.407, *I*^2^ = 0%; Figure 9 and Table 4). The high heterogeneity in the interoperative outcomes was attributed to one study [17] that measured intraoperative complications as “involuntary patient movements, such as diaphragm or abdominal wall contractions, and spontaneous breathing efforts or coughing” and therefore the control arm had 45 listed complications.

**Table 4.** Safety outcomes in the LPP group (*n *=* *280) and the SPP group (*n *=* *257)

Study	Intraoperative complications, *n* (%)		Postoperative complications, *n* (%)		Major postoperative complications, *n* (%)		Estimated blood loss, mL, mean ± SD	
	LPP	SPP	LPP	SPP	LPP	SPP	LPP	SPP
Albers *et al.* [14]	10 (11.2)	13 (14.6)	22 (24.7)	30 (33.7)	9 (10.1)	9 (10.1)	42 ± 85	42 ± 94
Celarier *et al.* [16]	5 (8.1)	2 (3.1)	10 (16.1)	19 (29.2)	5 (8.1)	3 (4.6)	NA	NA
Díaz-Cambronero *et al.* [17]	1 (1.2)	45 (55.6)	17 (20.0)	12 (14.8)	4 (4.7)	1 (1.2)	NA	NA
Cai *et al.* [15]	NA	NA	1 (2.3)	1 (4.5)	0 (0)	0 (0)	92.8 ± 27.1	87.5 ± 14.4

NA = not available, LPP = low-pressure pneumoperitoneum, SPP = standard-pressure pneumoperitoneum, SD = standard deviation.

### Adjustments in pneumoperitoneum

In three RCTs, changes in pneumoperitoneum levels were allowed at the surgeon’s request [14, 16, 17]. Albers *et al.* [14] had 12 (13.5%) requests of a 2 mmHg increase and 10 (11.2%) requests of a 4 mmHg increase in pressure in the LPP group versus 9 (10.1%) requests of a 2 mmHg increase and 4 (4.5%) requests of a 4 mmHg increase in pressure in the SPP group. Celarier *et al.* [16] had 9 (14.5%) requests of increased pressure to the standard levels in the LPP group and no requests of pressure increase in the SPP group with 23% and 3% of surgeons reporting “poor exposure” in each group, respectively. A total of 81% of the patients who had “poor exposure” were obese. Díaz-Cambronero *et al.* [17] reported that 20 (23.5%) cases in the LPP group and 7 (8.6%) cases in the SPP group had requests for increased pneumoperitoneum pressure; however, 80 (94%) patients in the LPP did not increase pressure high enough to cross over into the SPP group.

### Sensitivity analyses

#### Surgery for malignancy

Sensitivity analysis of the three RCTs [14, 16, 17] in which cancer was the indication for surgery in approximately ≥90% of cases performed revealed no significant differences in intraoperative complications (OR: 0.093, 95% CI: 0.001–6.570, *P *=* *0.274, *I*^2^ = 93.3%), postoperative complications (OR: 0.870, 95% CI: 0.531–1.427, *P *=* *0.582, *I*^2^ = 18%), or major complications (OR: 1.261, 95% CI: 0.547–2.906, *P *=* *0.586, *I*^2^ = 0). Statistical heterogeneity was minimized after sensitivity analysis as the *I*^2^ was reduced to 18% for postoperative complications and 0 for major complications. Additionally, the differences in operative time (WMD: −3.536, 95% CI: −14.904 to 7.832, *P *=* *0.542, *I*^2^ = 45%) and LOS (WMD: −3.536, 95% CI: −14.904 to 7.832, *P *=* *0.542, *I*^2^ = 45%) remained the same between the groups.

#### Laparoscopic surgery

A sensitivity analysis of the two RCTs [15, 17] that included only laparoscopic surgery showed no differences in postoperative complications (OR: 1.325, 95% CI: 0.613–2.866, *P *=* *0.474, *I*^2^ = 0), or major complications (OR: 2.425, 95% CI: 0.474–12.397, *P *=* *0.287, *I*^2^ = 0). Statistical heterogeneity was minimized after sensitivity analysis as the *I*^2^ was reduced to 0. A sensitivity analysis of operative time and LOS was not possible as they were reported by only one study [15].

#### Neuromuscular blockade

When studies were stratified by the standardization of NMB, two studies did not change this parameter between the LPP and SPP groups [15, 16]. When sensitivity analysis was performed using only these two studies, there remained no difference in postoperative complications (OR: 2.101, 95% CI: 0.949–4.654, *P *=* *0.067, *I*^2^ = 12%), or major complications (OR: 1.555, 95% CI: 0.404–5.992, *P *=* *0.521, *I*^2^ = 0). Statistical heterogeneity was minimized after sensitivity analysis as the *I*^2^ was reduced to 12% and 0. Additionally, differences in operative time (WMD: 4.446, 95% CI: −25.338 to 34.231, *P *=* *0.770, *I*^2^ = 76%) and LOS (WMD: −3.853, 95% CI: −11.688 to 3.981, *P *=* *0.335, *I*^2^ = 96%) remained the same between the two groups. Unfortunately, pain scores in the PACU and on POD1 could not be statistically pooled as only one study [16] reported both standardized NMB technique and pain scores. The authors did, however, report lower pain scores in PACU of LPP and SPP (2.5 ± 2.6 vs 3.5 ± 2.9), respectively, and similar scores on POD1 (2.2 ± 2.3 vs 2.4 ± 1.3).

#### Leave-one-out analysis

A leave-one-out meta-analysis showed no remarkable change in the odds of intraoperative and major postoperative complications, and mean difference of operative time, and LOS when each study was excluded. The odds of postoperative complications remained insignificant when each study was excluded except when the study by Díaz-Cambronero *et al.* [17] was excluded as the OR became 0.570 (95% CI: 0.342–0.951, *P *=* *0.031).

### Meta-regression

A meta-regression was performed to assess for factors associated with postoperative and major postoperative complications in patients undergoing an LPP strategy (Tables 5 and 6).

**Table 5.** Meta-regression for complications in low-pressure pneumoperitoneum (patient factors)

Variable	All complications		Major complications	
	Coefficient	*P*-value	Coefficient	*P*-value
Age	SE: 0.036	0.001*	SE: 0.010	0.021*
Male	SE: 0.006	<0.001*	SE: 0.002	0.043*
BMI	SE: 0.019	0.405	SE: −0.012	0.427
ASA II–IV	SE: 0.002	0.351	SE: −0.001	0.521

* Denotes significance.

BMI = body mass index, ASA = American Society of Anesthesiologists, SE = standard error.

**Table 6.** Meta-regression for complications in low-pressure pneumoperitoneum (surgical factors)

Variable	All complications		Major complications	
	Coefficient	*P*-value	Coefficient	*P*-value
Laparoscopic	SE: 0.011	0.060*	SE: 0.015	0.311
Robotic	SE: −0.006	0.232	SE: −0.013	0.238
Operative time	SE: 0.002	0.027*	SE: 0.001	0.010*
Intra-operative complications	SE: 0.05	0.434	SE: 0.006	0.160
Right sided resection	SE: 0.015	0.546	SE: −0.040	0.477
Left sided resection	SE: −0.0008	0.957	SE: −0.025	0.339
Subtotal/total colectomy	SE: 0.233	0.269	SE: 0.143	0.824

* Denotes significance.

SE = standard error.

The risk factors significantly associated with postoperative complications were age (SE: 0.036, *P *=* *0.001), male sex (SE: 0.006, *P *<* *0.001), and operative time (SE: 0.002, *P *=* *0.027), whereas BMI, ASA II–IV, and intraoperative complications were not associated with complications (all *P *>* *0.05). Only laparoscopic surgery was significantly associated with overall postoperative complications (SE: 0.011, *P *=* *0.060). Conversely, robotic surgery, right-sided colectomy, left-sided colectomy, or subtotal colectomy were not associated with complications (all *P *>* *0.05).

The risk factors significantly associated with major complications were age (SE: 0.010, *P *=* *0.021), male sex (SE: 0.002, *P *=* *0.043), and operative time (SE: 0.001, *P *=* *0.010), whereas BMI, ASA II–IV, and intraoperative complications were not associated with complications (all *P *>* *0.05). The site and approach of surgery were not associated with major complications (all *P *>* *0.05).

An additional meta-regression was performed to assess for factors associated with postoperative pain in the PACU and pain on POD1 (Supplementary Table 1). The site and approach of colectomy did not significantly alter the mean difference in pain scores between the two groups at either time point.

### Risk of bias and certainty of evidence

Three studies had a low risk of bias and one [3] had some concern of bias (Supplementary Figure 1). The certainty of evidence was high for pain score in PACU, pain score on POD1, postoperative complications, major postoperative complications, and blood loss, moderate for operative time, low for intraoperative complications, and very low for LOS (Supplementary Table 2).

## Discussion

The present meta-analysis found that the use of LPP was associated with lower pain scores in PACU and on POD1 with similar intraoperative and postoperative complications, operative time, blood loss, and LOS as compared with SPP. Three of the four included studies were conducted in Europe. While there is some high-quality evidence on the effect of LPP on the outcome of colorectal surgery, multi-center studies would be needed to increase the strength of the available evidence. Prior meta-analyses assessed the outcome of LPP in the context of cholecystectomy [8] and hysterectomy [7]. Both meta-analyses agree with the conclusion of our meta-analysis that LPP can decrease postoperative pain while not compromising the safety profile of the procedure. Our study sought to determine if the presumed benefits of LPP in other laparoscopic procedures can be reproduced in colorectal surgery.

The most important finding of our study is that LPP can decrease postoperative pain in minimally invasive colorectal surgery. This finding was based on a high degree of certainty with very low heterogeneity. However, it should be noted that despite the statistical significance of this finding, its clinical relevance is unclear since the magnitude of change in pain score was only one point on a 10-point scale for pain in PACU and one half of a point on POD1. It has been previously shown that LPP reduces analgesic consumption [18, 19], shoulder pain [20], and pain scores [19, 21] in other disciplines of minimally invasive surgery. The addition of deep NMB to an LPP strategy in hysterectomy, a common component of the LPP protocols utilized in the RCTs included in our analysis [14–17], has also been associated with reduced shoulder pain [22]. However, we do not have enough evidence to draw conclusions about long-term pain control or total analgesic consumption due to lack of reporting in the trials reviewed.

One common concern with the initiation of LPP protocols is the risk of decreasing safety while increasing operative times. Our meta-analysis refuted this concern. We demonstrated equivalent safety profiles in our analysis, concordant with multiple meta-analyses across other domains of surgery showing equivalent safety profiles [7, 23, 24]. In a meta-analysis involving cholecystectomy by Hua *et al.* [24], 22 RCTs were analyzed and the LPP group was shown to have a significantly longer operative time (WMD, 2.07 min; 95% CI, 1.09–3.05; *P* < 0.001; *I*^2^ = 26%). The opposite was found in a publication by El-Taji *et al.* [25] that analyzed four studies comparing peritoneum strategies in robotic prostatectomy and showed no difference in operative times (WMD −1.79; 95% CI, −15.96 to 12.38; *P *=* *0.8). The effect on operative time was again investigated in minimally invasive hysterectomy and was shown to be only 19 minutes longer, yet without attaining statistical significance (*P *=* *0.06) [26]. The heterogeneity of results on operative times could represent variation among surgical disciplines or the need for larger randomized trials on the topic.

In our study, three of the four RCTs allowed for changes in pneumoperitoneum pressures at the surgeon’s request [14, 16, 17]. The requested increase in pneumoperitoneum varied among surgeons and studies; however, requests for pressure increase were recorded in 14.5%–25% of cases in the LPP group and 9%–15% of cases in the SPP group. The rate at which the requested increase made the LPP group cross over into the same pressures as the SPP group varied from 5.9% to 14.5%. This range is very similar to the pressure for surgeons who began in the SPP group and requested increased pneumoperitoneum pressure. Finally, this is supported by the study on cholecystectomy by Hua *et al.*, which showed that there were more requests to increase pneumoperitoneum in the LPP group [24]. Although there was a higher rate of reporting “poor visibility” (23% vs 3%) in the LPP group [16], there were no differences in complications or operative times. These findings, in combination with the above findings, support the adaptability of the LPP approach based on surgeon preference with good compliance among surgeons even when given the option to increase pneumoperitoneum pressures.

To avoid as much confounding as possible, sensitivity analyses were conducted including studies that assessed surgery predominately for malignancy [14, 16, 17], studies that only utilized laparoscopic surgery [15, 17], and studies where the depth of NMB did not vary between groups [15, 16]. Surgery for malignant disease may be innately more complex with the addition of lymphadenectomy, possibility of an irradiated field, and broader margins among other factors. Ilyas *et al.* [27] showed that there was no difference in overall complication rates between sigmoidectomy for benign versus malignant disease. In this current study, when benign disease was excluded, we did not find any difference in safety, operative time, or LOS with an LPP strategy. Additionally, in the meta-analysis by Wang *et al.* [28], it was shown that robotic surgery had a longer operative time when compared with laparoscopic surgery. When analysis was limited to studies that included laparoscopic surgery only, we again did not find any difference in safety, operative time, or LOS with an LPP strategy in our study. Finally, depth of NMB has been investigated in colorectal surgery as a method of improving visualization and limiting complications. Moderate NMB combined with a transverse abdominal plane block was shown to have similar visualization and outcomes to deep NMB without transverse abdominal plane block [29]. Additionally, when investigating LPP strategies, depth of NMB was shown to have a trend toward lower pain and complications without reaching statistical significance [30]. Our results of similar outcomes among patients with consistent NMB depth support the above conclusion that the effect could be related to the depth of blockade rather than the pneumoperitoneal pressure.

When we excluded the study by Díaz-Cambronero *et al.* [17], the odds of postoperative complications were decreased when LPP was used. This finding makes sense, given that this particular study was the only one that reported a higher rate of postoperative complications in the LPP group. This discrepancy could be attributed to the fact that the LPP group in their study had the most variation in the pneumoperitoneum used. Their protocol instilled 15 mmHg pressure during trocar insertion for a minimum of 5 minutes followed by progressive decreases in pneumoperitoneum at the discretion of the surgeon. However, the reason for this variability in outcome is unclear.

The meta-regression analysis showed that older and male patients and patients with extended operative times may have an increased risk of overall and major postoperative complications when LPP is employed. All of these factors are in line with the previous risk models in the published literature [31]. Additionally, male patients have been shown to have both a higher rate of postoperative complications [32] and anastomotic leak [33] within the field of colorectal surgery. Increasing ASA status was not associated with more complications in this analysis. This could be related to the high rate of cardiac comorbidities that play into ASA status and the improved systolic blood pressure, heart rate, and end tidal CO_2_ that have been shown in LPP strategies in cholecystectomy [34]. Furthermore, our meta-regression analysis did not show site or approach of surgery to have an association with pain scores in the PACU or on POD1. This implies that the beneficial effect of this strategy is independent of operative approach or site. However, the fact that these data are dervied from only two studies limits the generalizability and rigor of these findings.

Finally, laparoscopic surgery was associated with an increased risk of overall complications but not major complications in LPP. The same effect was not observed in robotic surgery. The site of resection was not associated with an increased risk of overall or major complications. Robotic surgery has been shown to have a shorter learning curve and lower conversion rate than laparoscopic surgery [35]. Additionally, it has been shown to have a shorter LOS and lower mortality [36]. However, other studies have not found the same reductions in complications over laparoscopic surgery with increased costs and surgical time [37]. The current study found increased overall complications with laparoscopic surgery but no effect on LOS or any of our other outcomes. For this reason, LPP may not be suitable for revisional colorectal surgery or in challenging cancer cases that are performed laparoscopically.

A strength of the current meta-analysis and meta-regression is the inclusion of only RCTs. This introduces a low risk of bias, producing a moderate to high certainty of evidence in most assessed outcomes. However, the study is limited by the small number of included studies and lack of reporting of some parameters in some trials. The high statistical and clinical heterogeneity among the studies was another limitation. We addressed this deficit by performing sensitivity analyses that were able to minimize the heterogeneity. The consistency of findings from the overall analysis after sensitivity analyses may imply a marginal effect of the aforementioned heterogeneity. Additionally, inconsistency in terms of assessment of postoperative pain was noted across studies. The confounding of NMB on pain scores could not be addressed as only one study [16] kept NMB constant between groups and reported pain scores. This calls for a standardized method for pain assessment after laparoscopic colorectal surgery. The low and very low levels of evidence for intraoperative complications and LOS can be explained by the variation in how intraoperative complications [17] were defined and a very large outlier in LOS [16], respectively. Overall, despite the small number of studies included and limited data on pain assessment at different time points, the present meta-analysis does add to the literature by showing the safety of low intra-abdominal pressure and highlighting the gaps in the literature that warrants a multi-center large RCT. It should be noted, however, that the results of meta-regression analyses should be interpreted with caution since these types of analyses have low statistical power. Therefore, the findings of our meta-regression analyses need to be ascertained in future prospective studies.

## Conclusions

LPP is associated with lower pain scores in PACU and on POD1 with similar operative times, LOS, and safety profile to SPP in colorectal surgery. Although LPP was not associated with higher complications, patients with advanced age, male sex, undergoing laparoscopic compared with robotic surgery, and those with longer operative times may be at risk of increased complications when this strategy is utilized.

## Supplementary Material

goae052_Supplementary_Data
