# Supplementary material for: Low-pressure versus standard-pressure pneumoperitoneum in minimally invasive colorectal surgery: a systematic review, meta-analysis, and meta-regression analysis
Source: Gastroenterol Rep (Oxf). 2024 Jul 19;12:goae052. doi: 10.1093/gastro/goae052 (PMC11259227; doi:10.1093/gastro/goae052)
Supplement: goae052_Supplementary_Data [file goae052_supplementary_data.zip › Supplementary Table 2[AU].docx]

**Supplementary Table 1: GRADE Analysis of Certainty of Evidence**

**Question:** Low pressure pneumoperitoneum compared to standard pressure pneumoperitoneum for colorectal surgery

| **Certainty assessment** | | | | | | | **№ of patients** | | **Effect** | | **Certainty** | **Importance** |
| --- | --- | --- | --- | --- | --- | --- | --- | --- | --- | --- | --- | --- |
| **№ of studies** | **Study design** | **Risk of bias** | **Inconsistency** | **Indirectness** | **Imprecision** | **Other considerations** | **Low pressure pneumoperitoneum** | **Standard pressure pneumoperitoneum** | **Relative** **(95% CI)** | **Absolute** **(95% CI)** |  |  |
| **Pain Score in PACU** | | | | | | | | | | | | |
| 2 | Randomized trials | Not serious | Not serious | Not serious | Not serious | None | 151 | 154 | - | MD **1.06 lower**  **(1.65 lower to 0.47 lower)** | ⨁⨁⨁⨁ High | CRITICAL |
| **Pain Score POD 1** | | | | | | | | | | | | |
| 2 | Randomized trials | Not serious | Not serious | Not serious | Not serious | None | 151 | 154 | - | MD **0.49 lower**  **(0.91 lower to 0.07 lower)** | ⨁⨁⨁⨁  High | CRITICAL |
| **Postoperative Complications** | | | | | | | | | | | | |
| 4 | Randomized trials | Not serious | Not serious | Not serious | Not serious | None | 50/280 (17.9%) | 62/257 (24.1%) | **OR 0.74** **(0.49 to 1.14)** | **51 fewer per 1,000** **(from 106 fewer to 25 more)** | ⨁⨁⨁⨁ High | CRITICAL |
| **Major Postoperative Complication** | | | | | | | | | | | | |
| 4 | Randomized trials | Not serious | Not serious | Not serious | Not serious | None | 18/280 (6.4%) | 13/257 (5.1%) | **OR 1.43** **(0.68 to 3.01)** | **20 more per 1,000** **(from 16 fewer to 88 more)** | ⨁⨁⨁⨁ High | CRITICAL |
| **Operative Time** | | | | | | | | | | | | |
| 3 | Randomized trials | Not serious | Serious^c^ | Not serious | Not serious | None | 195 | 176 | - | MD **1.99 minutes higher**  (12.97 lower to 16.95 higher) | ⨁⨁⨁◯ Moderate | IMPORTANT |
| **Length of Stay** | | | | | | | | | | | | |
| 3 | Randomized trials | Not serious | Very serious^a^ | Serious^b^ | Not serious | None | 195 | 196 | - | MD **2.3 days lower**  (7.68 lower to 3.07 higher) | ⨁◯◯◯ Very low | IMPORTANT |
| **Blood Loss** | | | | | | | | | | | | |
| 2 | Randomized trials | Not serious | Not serious | Not serious | Not serious | None | 133 | 111 | - | MD **4.63 mL higher**  **(4.73 lower to 13.99 higher)** | ⨁⨁⨁⨁ High | IMPORTANT |

**CI:** confidence interval; **OR:** odds ratio

#### **Explanations**

a. One study (Celarier et al.) had a significant outlier of 43 days in the control group.

b. Outcome not directly related to intervention

c. I^2 value > 50% and < 75%

d. One study (Diaz-Cambronero et al) measured intraoperative complications as "involuntary patient movements, such as diaphragm or abdominal wall contractions, and spontaneous breathing efforts or coughing” and therefore the control arm had 45 listed complications.
