# Supplementary material for: Low-pressure versus standard-pressure pneumoperitoneum in minimally invasive colorectal surgery: a systematic review, meta-analysis, and meta-regression analysis
Source: Gastroenterol Rep (Oxf). 2024 Jul 19;12:goae052. doi: 10.1093/gastro/goae052 (PMC11259227; doi:10.1093/gastro/goae052)
Supplement: goae052_Supplementary_Data [file goae052_supplementary_data.zip › Supplementary Table 1[AU].docx]

**Supplementary Table 1: Meta-regression for Pain in Low Pressure Pneumoperitoneum: Surgical Factors**

|  | **Pain in PACU** | | **Pain on POD1** | |
| --- | --- | --- | --- | --- |
| **Variable** | **Coefficient** | **P-value** | **Coefficient** | **P-value** |
| Laparoscopic | SE: 0.05 | 0.872 | SE: 0.195 | 0.417 |
| Robotic | SE: -0.002 | 0.872 | SE: -0.007 | 0.417 |
| Right Sided Resection | SE: -0.006 | 0.872 | SE: -0.024 | 0.417 |
| Left Sided Resection | SE: -0.003 | 0.872 | SE: -0.012 | 0.417 |
| Subtotal / Total Colectomy | SE: -0.1 | 0.872 | SE: -0.39 | 0.417 |

PACU, post anesthesia care unit; POD, postoperative day
