# Supplementary figures and images for: Low-pressure versus standard-pressure pneumoperitoneum in minimally invasive colorectal surgery: a systematic review, meta-analysis, and meta-regression analysis
Source: Gastroenterol Rep (Oxf). 2024 Jul 19;12:goae052. doi: 10.1093/gastro/goae052 (PMC11259227; doi:10.1093/gastro/goae052)

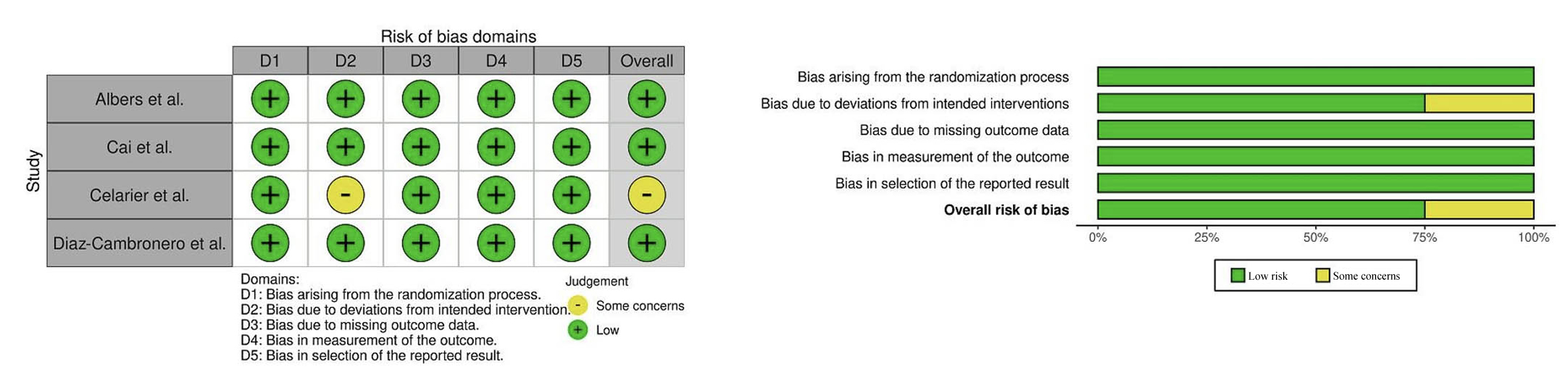

Supplement: goae052_Supplementary_Data [file goae052_supplementary_data.zip › Supplementary Fig 1 final version.jpg]
